# PROSOCIALITY AND SUBSEQUENT COGNITIVE HEALTH: A PROSPECTIVE COHORT STUDY

**DOI:** 10.1093/geroni/igae098.2135

**Published:** 2024-12-31

**Authors:** Justin Farmer, Lucía Macchia, Jessica Gong, Andrew Steptoe, Feifei Bu, Laura Kubzansky

**Affiliations:** Harvard T.H. Chan School of Public Health, Somerville, Massachusetts, United States; City College London, London, England, United Kingdom; University College London, London, England, United Kingdom; University College London, London, England, United Kingdom; University College London, London, England, United Kingdom; Harvard T. H. Chan School of Public Health, Boston, Massachusetts, United States

## Abstract

Specific prosocial behaviors, including volunteering and caregiving, have been linked to better maintenance of cognitive health in older adults. However, limited epidemiologic work has examined the relationship between one’s cognitive-emotional orientation toward prosociality generally and these same health outcomes. This study uses data on 7844 participants from the English Longitudinal Study of Ageing (ELSA) to test the hypothesis that higher versus lower prosociality is associated with higher maintenance of cognitive health in adults 50 years or older. We derived a 9-item prosociality scale using items from well-validated measures of self-reported altruism and collectivism assessed in ELSA. Linear mixed-effects models were used to examine the relationship between this measure and changes in executive function and verbal memory over an 11-year period. Cox proportional hazards models were then used to compare time to dementia based on level of prosociality. Results from both longitudinal and survival analyses suggest that higher prosociality is associated with maintaining better cognitive health even after controlling for sociodemographics (e.g., age, education, wealth) and baseline health characteristics such as depression and cardiovascular disease. Comparing across tertiles, participants with high versus low prosociality had 24% slower decline in verbal memory and 55% slower decline in executive function. This aligned with a 35% reduced hazard of dementia during this same period for those with high vs. low prosociality. These results suggests that prosociality, beyond formal volunteering, is an important modifiable risk factor for improving later life cognitive function on an individual and population level and should therefore be further examined.

